# Respiratory Function in Voluntary Participating Patagonia Sea Lions (*Otaria flavescens*) in Sternal Recumbency

**DOI:** 10.3389/fphys.2016.00528

**Published:** 2016-11-16

**Authors:** Andreas Fahlman, Johnny Madigan

**Affiliations:** ^1^Fundación Oceanografic de la Comunidad ValencianaValencia, Spain; ^2^Department of Life Sciences, Texas A&M University-Corpus ChristiCorpus Christi, TX, USA; ^3^Dolphin AdventureNuevo Vallarta, Mexico

**Keywords:** tidal volume, breath duration, diving physiology, respiratory flow rate, lung compliance

## Abstract

We measured esophageal pressures (*n* = 4), respiratory flow rates (*n* = 5), and expired O_2_ and CO_2_ (*n* = 4) in five adult Patagonia sea lions (*Otaria flavescens*, body mass range 94.3–286.0 kg) during voluntary breaths while laying down out of water. The data were used to estimate the dynamic specific lung compliance (sCL), the O_2_ consumption rate ($\dot{V}$*O*_2_) and CO_2_ production rates ($\dot{V}$*CO*_2_) during rest. Our results indicate that the resting tidal volume in Patagonia sea lions is approximately 47–73% of the estimated total lung capacity. The esophageal pressures indicated that expiration is passive during voluntary breaths. The average sCL of sea lions was 0.41 ± 0.11 cmH_2_O^−1^, which is similar to those measured in anesthetized sea lions and awake cetaceans, and significantly higher as compared to humans (0.08 cmH_2_O^−1^). The average estimated $\dot{V}$*O*_2_ and $\dot{V}$*CO*_2_ using breath-by-breath respirometry were 1.023 ± 0.327 L O_2_ min^−1^ (range: 0.695–1.514 L O_2_ min^−1^) and 0.777 ± 0.318 L CO_2_ min^−1^, (range: 0.510–1.235 L CO_2_ min^−1^), respectively, which is similar to previously published metabolic measurements from California and Steller sea lions using conventional flow-through respirometry. Our data provide end-tidal gas composition and offer novel data for respiratory physiology in pinnipeds, which may be important for clinical medicine and conservation efforts.

## Introduction

In 1940, Per Scholander (1940) published an article that described the unusual properties of marine mammal respiratory systems. Scholander suggested that the highly compliant lung and rib cage would easily compress, the air being shunted into the rigid upper airway. This hypothesis has remained a central tenet in marine mammal diving physiology, with the assumption that this physiological trait will reduce uptake of N_2_ and the risk of decompression sickness (DCS). A few studies have attempted to determine how pressure affects gas exchange in forced or freely diving marine mammals. These studies have assessed alveolar compression either directly by quantifying the depth related pulmonary shunt (Kooyman and Sinnett, 1982; McDonald and Ponganis, 2012), or indirectly by measuring N_2_ uptake in arterial and venous blood during a dive (Kooyman et al., 1972; Falke et al., 1985) or measuring N_2_ removal from the muscle following a series of repeated dives (Ridgway and Howard, 1979; Houser et al., 2010). As the estimated depth at which alveolar collapse occurs differs considerably between studies, Bostrom et al. (2008) developed a mathematical model in an attempt to understand lung compression in marine mammals. The objective was to create a theoretical frame-work that could be used to predict the air volumes of the various compartments of the respiratory system to the limit of collapse. The results from the model suggested that the diving lung volume, the relative size of the upper (conducting airways) and lower (alveoli) airways, and the structural properties (compliance) of the upper and lower airways were important in determining the depth at which the alveoli collapse and gas exchange ceases (Bostrom et al., 2008). The model output was compared to available data for collapse depth in different species (Kooyman et al., 1970, 1972; Ridgway and Howard, 1979; Kooyman and Sinnett, 1982; Falke et al., 1985), and it was concluded that behavioral (diving lung volume) and structural (lung and dead space compliance) variations between species could account for the observed species differences (Bostrom et al., 2008; Fahlman et al., 2009).

Static pressure-volume loops are commonly used to measure the physical properties (compliance) of the respiratory system. Published data exist on excised lungs for several terrestrial species, but only a few measurements have been made for marine mammals (Denison et al., 1971; Piscitelli et al., 2010; Fahlman et al., 2011; Moore et al., 2014). A recently published theoretical study compared estimated blood and tissue gas tensions (O_2_, CO_2_, and N_2_) using species-specific respiratory compliance estimates or those previously published from terrestrial mammals (Hodanbosi et al., 2016). The results showed that the blood and tissue PN_2_ levels were significantly lower using the species-specific compliance estimates, while there were little or no differences for blood and tissue PCO_2_ or PO_2_ levels. It is therefore difficult to assess the accuracy of the model output without species-specific respiratory compliance estimates. In addition, common indices of respiratory capacity and mechanics, e.g., lung compliance, inspiratory and expiratory tidal volume, flow-rate, and duration, exist for a limited number of marine mammals of different age classes (Parker, 1922, 1932; Scholander and Irving, 1941; Robin et al., 1963; Olsen et al., 1969; Kooyman et al., 1971; Leith, 1976; Gallivan, 1981; Kooyman and Cornell, 1981; Gallivan et al., 1986; Bergey and Baier, 1987; Reed et al., 1994, 2000; Fahlman et al., 2015). Only by acquiring comparable data from multiple species, can one begin to understand the level of confidence with which one can predict such parameters for species where we have little or no data. The present study was undertaken to extend the current knowledge of the respiratory physiology and mechanical properties of Patagonia sea lions (*Otaria flavescens*) participating voluntarily. In addition, we also collected end-expiratory gases and estimated breath-by-breath gas exchange. Our results indicate that the metabolic demand and respiratory physiology of Patagonia sea lions are similar to other pinniped species.

## Materials and methods

### Animals and morphometrics

Five Patagonia sea lions (*O. flavescens*), 3 females and 2 males, managed under human care participated under voluntary control (Table 1). All work was approved by the IACUC at Texas A&M University—Corpus Christi (TAMUCC-IACUC AUP # 04-11).

**Table 1:** **Animal ID, sex [female (F)/male(M)] age (years), body mass (*M*_*b*_, kg), rate of O_2_ consumption ($\dot{V}$*O*_2_, superscript number is the number of repeated measurements of at least 3 min), and CO_2_ production ($\dot{V}$*CO*_2_, number of measurements are the same as for $\dot{V}$*O*_2_), breathing frequency with mask on (*f*_*Rm*_, breaths min^−1^) or during a 5 min focal observation (*f*_*Rf*_), and lung compliance (C_*L*_) of 5 Patagonia sea lions (*Otaria flavescens*) participating in the study**.

**Animal ID**	**Sex**	**Age**	***M*_b_**	**$\dot{V}$*O*_2_**	**$\dot{V}$*CO*_2_**	***f*_Rm_**	***f*_Rf_**	**C_L_**
		**Yr**	**(kg)**	**(L min^−1^)**	**(L min^−1^)**	**bpm**		**L cmH_2_O^−1^**
Sayula	F	14	113.0±4.2	0.695±0.162^11^	0.510±0.145	4.6±1.8^9^	4.1±0.3^3^	0.22±0.08^50^
Jenny	F	14	100.5±2.1	0.840±0.234^10^	0.637±0.194	5.0±2.1^10^	8.3±0.2^3^	0.23±0.06^80^
Rex	M	5	143.5±4.9	1.514±0.361^14^	1.235±0.169	3.8±0.9^8^	4.1±0.2^3^	0.49±0.16^82^
Mara	F	14	94.3±4.6	0.881±0.123^7^	0.729±0.209	5.1±1.2^9^	4.6±1.3^3^	0.31±0.12^180^
Nano	M	15	286	N/A	N/A	3.3±0.9^3^	3.6±0.7^3^	N/A

Superscript numbers is the number of repeated measurements for that variable.

Each animal was weighed (±0.5 kg) before the start of each procedure (Table 1).

### Flow measurements

Ventilatory flow-rates were measured using a pneumotachometer (3813 series, 0–800 l min^−1^, Hans-Rudolph Inc., Shawnee, KS) placed inside a modified anesthesia face-mask. The maximum dead-space of the mask was 200 mL, and varied slightly depending on how much of the snout was placed inside the mask. The pneumotachometer was connected to a differential pressure transducer (MPX-2.5 mbar type 339/2, Harvard apparatus, Holliston, MA) via a 310 cm length of 2 mm I.D, firm walled, flexible tubing. The pneumotachometer was calibrated for linearity and flow using a 7 L calibration syringe (Series 4900, Hans-Rudolph Inc., Shawnee, KS) immediately before and after each trial, through a series of pump cycles at various flow rates. The pump cycles allowed the relationship between differential pressure and flows for the expiratory and inspiratory phases to be determined. To avoid spurious peaks, the reported maximal inspiratory and expiratory flows are the average flows over 20 ms; 10 ms on either side of the maximal recorded inspiratory or expiratory flow.

### Airway and esophageal pressures

**Figure 1:**
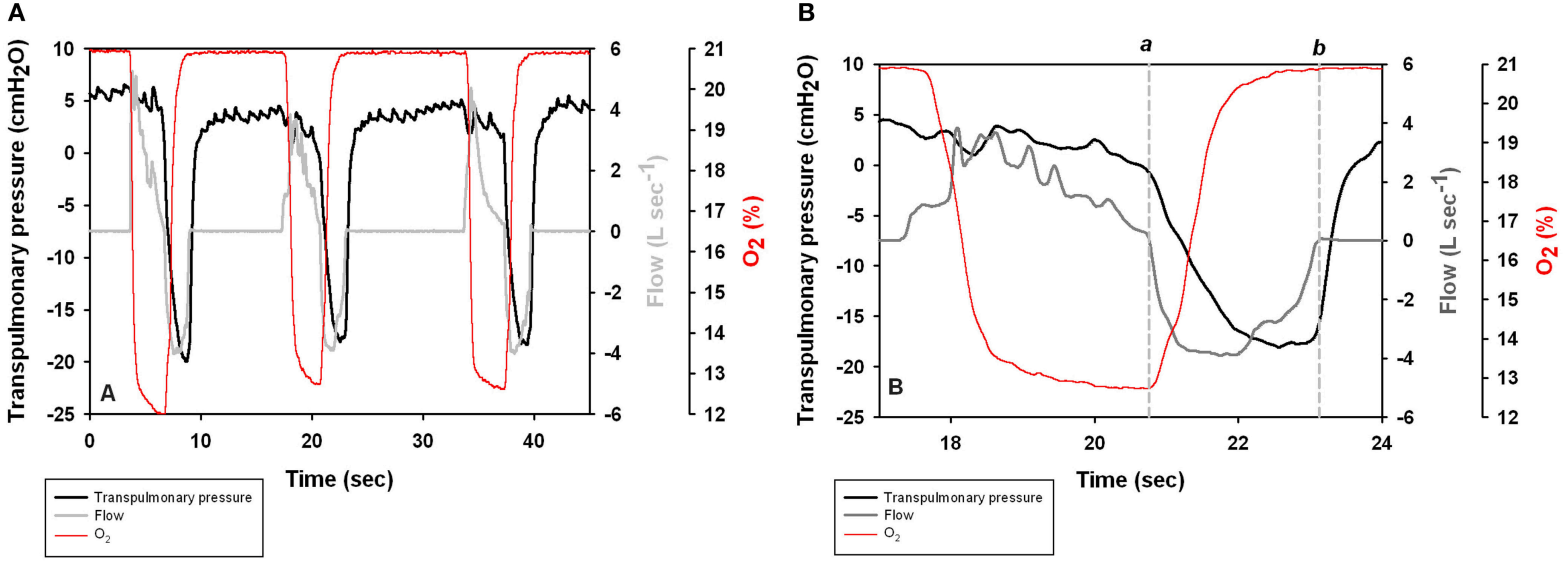
**(A)** Transpulmonary pressure (P_tp_ = P_ao_–P_eso_) pressure, respiratory flow, and O_2_ content during three voluntary breaths in a Patagonia sea lion. Positive values represent exhalation. **(B)** Expanded scale shows the spontaneous breath with times of 0 flow (vertical lines a and b) used to estimate lung compliance.

Airway pressure (P_aw_) was measured via a sample port immediately above the nostrils connected to a differential pressure transducer (MPX-100 mbar type 339/2, Harvard apparatus, Harvard Apparatus, Holliston, MA). Esophageal pressure (P_eso_) was measured using an esophageal balloon catheter (47–9005, Cooper Surgical, Trumbull, CT) connected to a differential pressure transducer (MPX-100 mbar type 339/2, Harvard apparatus, Holliston, MA) by a 288 cm length of 2 mm I.D., firm walled, flexible tubing, through a 3 way stopcock. The balloon catheter was manually inserted into the esophagus to the approximate level of the heart, and inflated with 1.0 ml of air (Figure 1). Reference pressure for both P_ao_ and P_eso_ was ambient atmospheric pressure (P_amb_).

### Data acquisition of differential pressures

Differential pressure transducers were connected to an amplifier (Tam-A, Harvard apparatus, Holliston, MA). The data from the transducers were captured at 400 Hz using a data acquisition system (Powerlab 8/35, ADInstruments, Colorado Springs, CO), and displayed on a laptop computer running LabChart (v. 8.1, ADInstruments, Colorado Springs, CO). All differential pressure transducers were zeroed immediately before each trial.

### Dynamic responses

The dynamic response of the system configured as described above was evaluated for a step response by a balloon deflation test. Popping an inflated balloon with a needle provided an immediate step change in pressure. The time constant (τ) was estimated as the time to 50% pressure reduction, or τ_1/2_. The dynamic constant was 7 ms for the pneumotachometer pressure line and 40 ms for the esophageal catheter.

### Respiratory gas composition

Respiratory gasses were subsampled via a port in the pneumotachometer and passed through a 310 cm length of 2 mm I.D., firm walled, flexible tubing and a 30 cm length of 1.5 mm i.d. Nafion tubing, to fast-response O_2_ and CO_2_ analyzers (ML206, Harvard Apparatus, Holliston, MA, USA) at a flow rate of 200 ml min^−1^. The gas analyzers were connected to the data acquisition system and sampled at 400 Hz. The gas analyzers were calibrated before and after the experiment using a commercial mixture of 5% O_2_, 5% CO_2_, and 90% N_2_, certified accurate to at least 0.01%. (Gasco, Oldsmar, FL. Prod#17L-340). Mean daily air temperature, humidity, and ambient pressure were 29.7 ± 2.9°C (range 20.1–33.6°C), 89.9 ± 13.8% (range 57–100%), and 101.0 ± 0.3 kPa (range 100.7–101.4 kPa, *n* = 32 trials).

### Lung compliance

Lung compliance (C_L_ = VT • Δ${\text{P}}_{\text{tp}}^{-1}$, L •cmH_2_O^−1^) was estimated as the inspiratory tidal volume divided by the change in transpulmonary pressure (ΔP_tp_ = ΔP_aw_ − ΔP_eso_), measured at zero flow (Olsen et al., 1969; Fahlman et al., 2015). The esophageal pressure trace was phase corrected by 40 ms (the dynamic response time of the esophageal catheter system), and P_eso_ was determined at the points of zero flow of the expiratory phase and inspiratory phases (a and b, respectively, in Figure 2C in Fahlman et al., 2015).

### Metabolic rates

The respiratory gas data were phase corrected to account for the delay caused by the flow in the sample line. The expiratory flow-rate and expired O_2_ content was multiplied to calculate the instantaneous $\dot{V}$*O*_2_. The instantaneous $\dot{V}$*O*_2_ was integrated over each breath to yield the total volume of O_2_ exchanged during each breath. The volume was summed for each trial period and divided by the duration of the trial to provide an estimate of the oxygen consumption rate for that time period.

To account for differences in body mass, $\dot{V}$*O*_2_ was converted to mass-corrected metabolic rate (s$\dot{V}$*O*_2_) using the previously predicted mass exponent for Steller sea lions (body mass^0.6^; McPhee et al., 2003).

### Data processing and statistical analysis

All gas volumes were converted to standard temperature pressure dry (STPD, Quanjer et al., 1993). Exhaled air was assumed saturated at 37°C, inhaled air volume was corrected for ambient temperature and relative humidity.

Metabolic data are reported as the average O_2_ consumption rate for a duration of calm continuous breathing for at least 3 min. Compliance data are reported as the average for all breaths in a trial (Table 1). The relationship between a dependent variable and experimental covariates was analyzed using linear-mixed effects models (lme, R: A Language and Environment for Statistical Computing, R Foundation for Statistical Computing, version 3.1.0, 2014). The individual animal was treated as a random effect, which accounted for the correlation between repeated measurements on the same individual (Littell et al., 1998). Initially, variables were selected for inclusion in a multivariate model if the univariate analysis had a *p* < 0.2 (Wald's test). Best models were chosen by the Akaike information criterion (AIC) against nested models and significance determined using the Likelihood ratio test (LRT). In this study *P* ≤ 0.05 were considered as significant and *P* ≤ 0.1 were considered a trend. Data are presented as the mean ± standard deviation (*SD*), unless otherwise stated.

## Results

A total of 30 experimental sessions were conducted in May and October 2015. The sex, age, and average during the measurement are reported in Table 1.

### Lung function

Figure 1A shows transpulmonary pressure, respiratory flow, and O_2_ concentration during voluntary breaths. The data for the second breath is presented in Figure 1B to illustrate the point used to calculate C_L_. Most breaths began with an exhalation followed by an inhalation.

**Figure 2:**
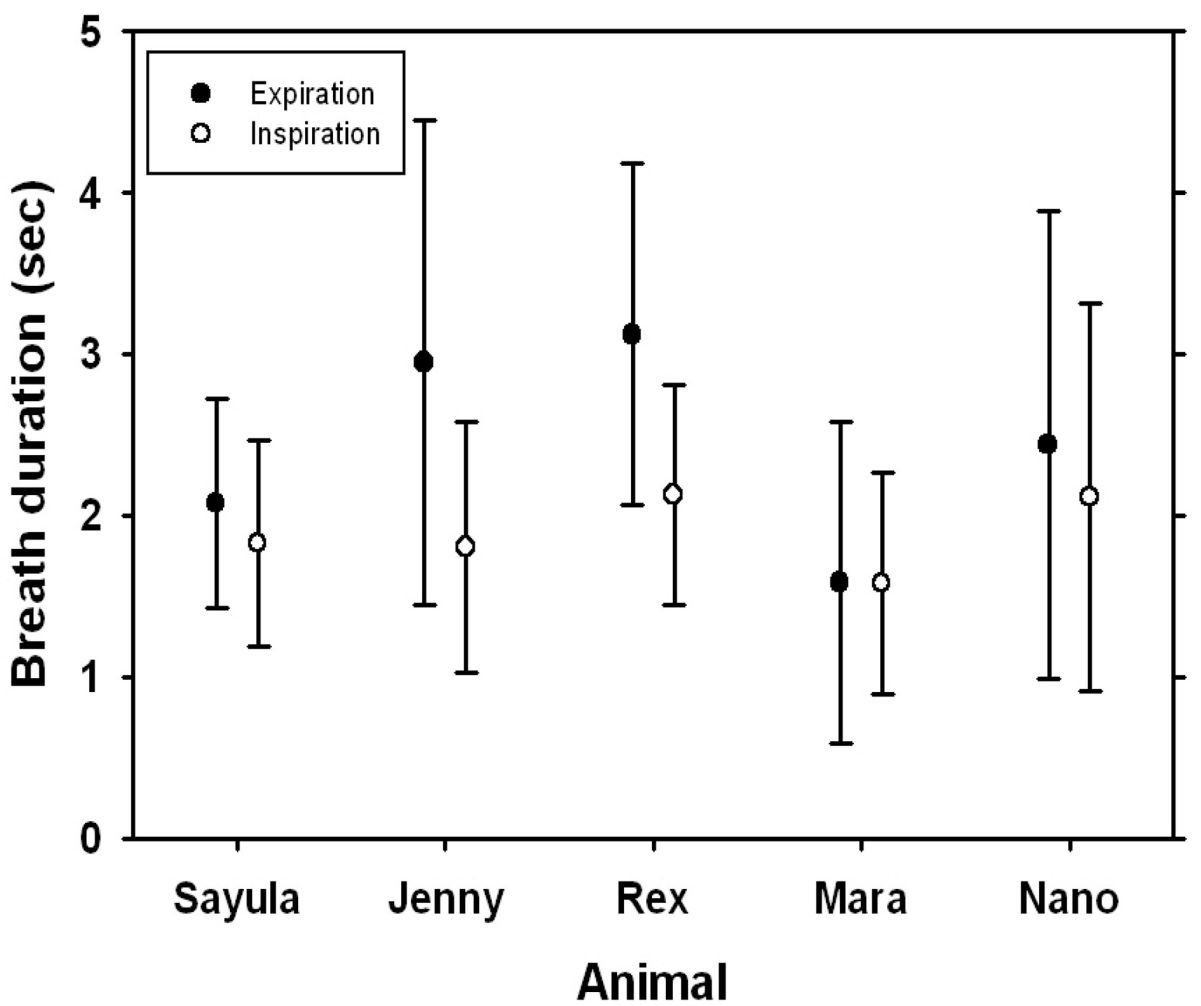
**Average (±*SD*) inspiratory and expiratory breath duration for five Patagonia sea lions**.

The average (±*SD*) expiratory, and inspiratory tidal volumes, respiratory flow rates, and lung compliance for a total of 874 breaths are reported in Table 1. The average respiratory frequency during trials was 4.4 ± 0.8 breaths min^−1^ (Table 1, range: 2.3–8.8), which was not significantly different (*P* > 0.2, paired *t*-test) from values observed during visual follows of animals observed in their enclosure out of water during free time (4.9 ± 1.9 breaths min^−1^). The respiratory frequency was significantly higher in the males as compared with the females (*P* < 0.01, paired *t*-test). The duration of the expiratory phase (2.43 ± 0.63 s, Figure 2) was significantly longer than that of the inspiratory phase (1.89 ± 0.23 s, *P* < 0.01, AIC_null_ = 4047, AIC_breath−type_ = 3948).

**Figure 3:**
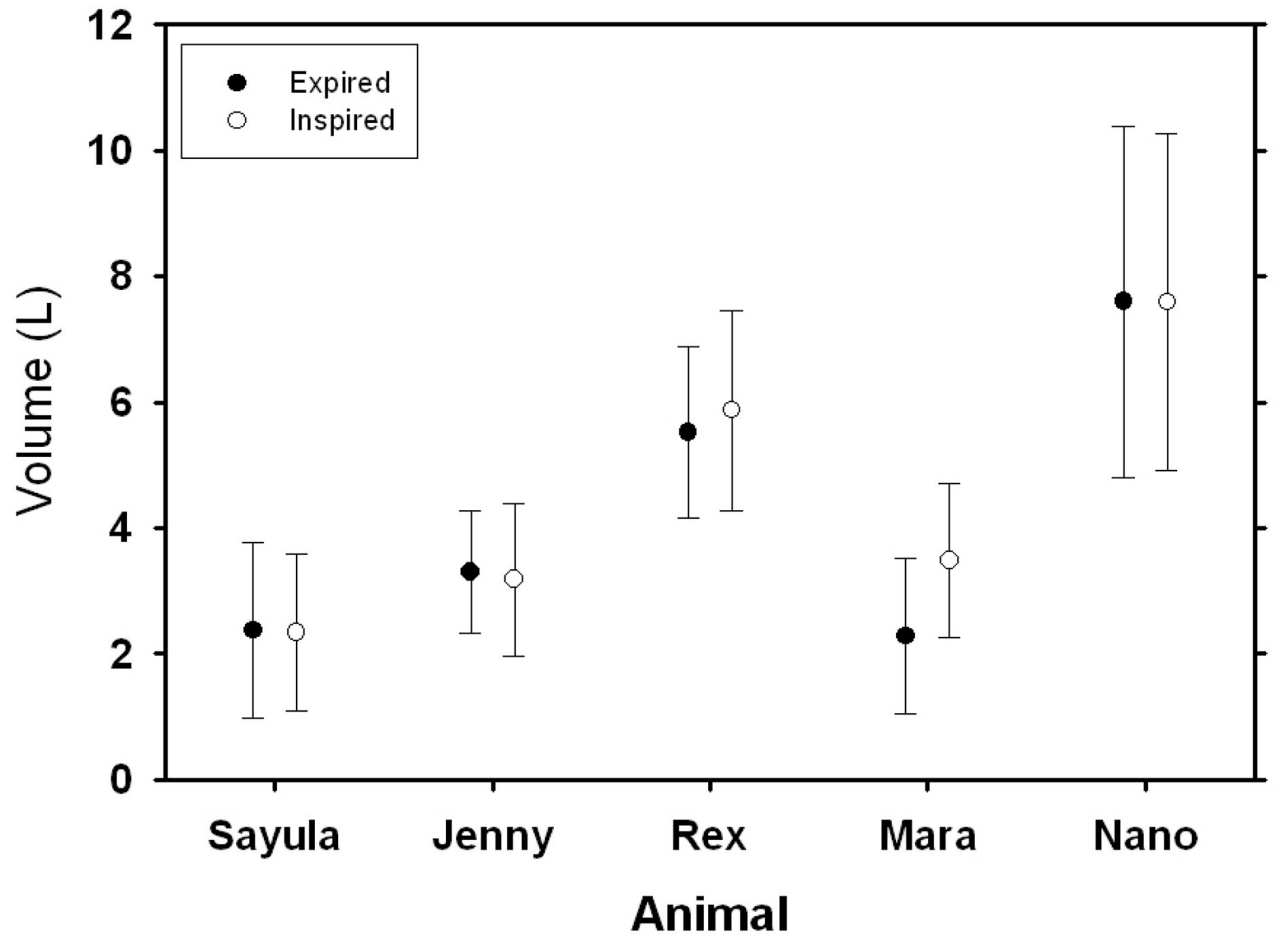
**Average (±*SD*) inspiratory and expiratory tidal volumes for five Patagonia sea lions**.

There was no difference in average (±*SD*) inspiratory volume (4.49 ± 2.17 L, Figure 3) as compared with the expiratory volume (4.21 ± 2.30 L, *P* > 0.7, AIC_null_ = 5411, AIC_breath−type_ = 5412). The tidal volume did not differ between animals (*P* > 0.1). The largest tidal volume recorded during this study was 11.4 l for Nano (41 mL kg^−1^), and the largest mass-specific tidal volume was measured for the smallest sea lion (Mara, 69 mL kg^−1^). The tidal volumes ranged from 47 to 73% of the estimated total lung capacity, based on models from excised lungs from a number of marine mammals species (${\text{TLC}}_{\text{est}}=0.135M_{\text{b}}^{0.92}$, Kooyman, 1973; Fahlman et al., 2011).

**Figure 4:**
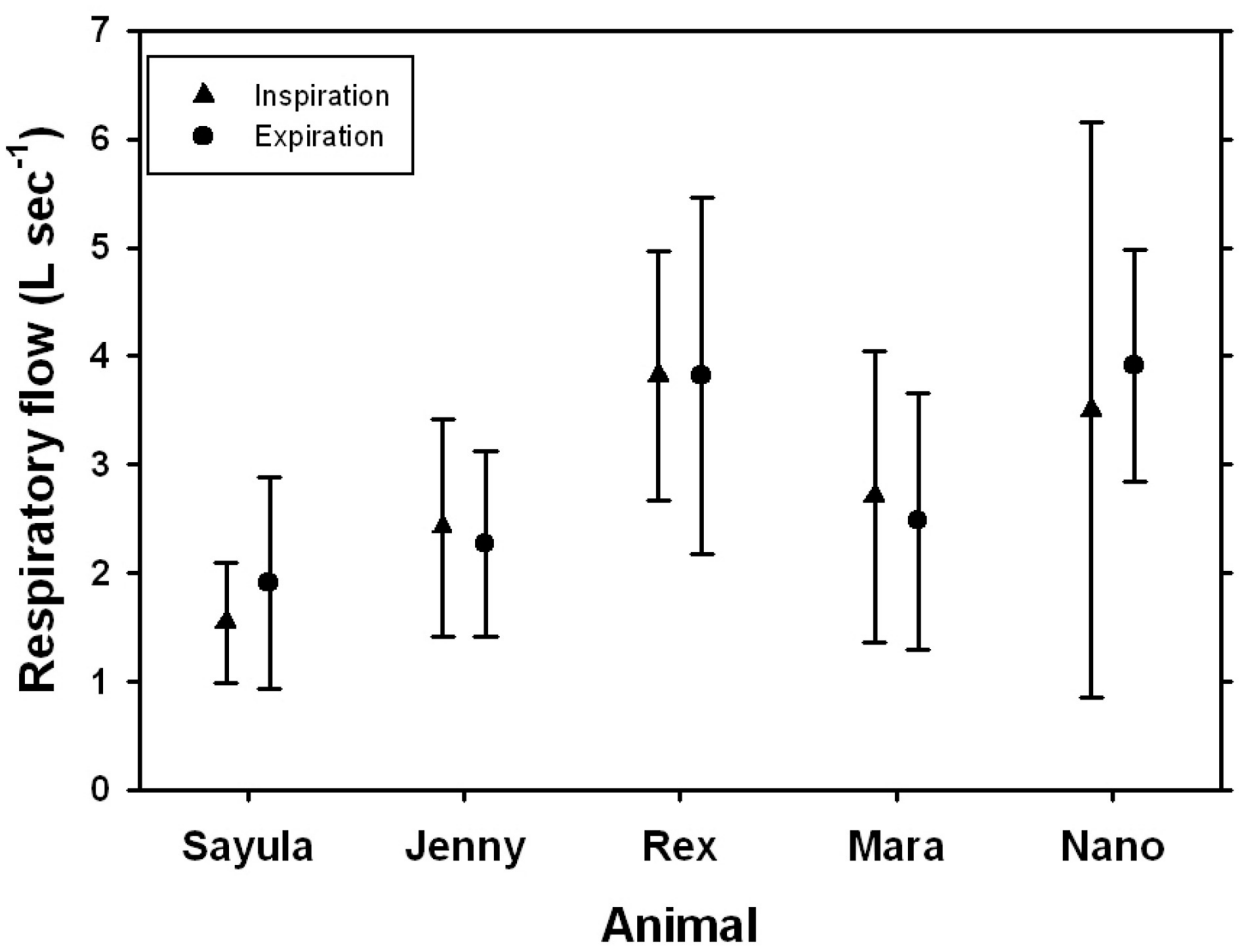
**Average (±*SD*) inspiratory and expiratory flow for five Patagonia sea lions**.

The average respiratory flows were similar for expiration and inspiration (Figure 4). Peak inspiratory flow of spontaneous breaths ranged from 0.4 to 7.0 L s^−1^ while peak expiratory flow ranged from 0.5 to 9.1 L s^−1^. When the peak inspiratory or expiratory flows were expressed as a fraction of TLC_est_ (*f*_TLC_ = maximum flow divided by TLC_est_), the maximum inspiratory flow ranged from 0.14 to 0.31 *f*_TLC_ s^−1^, while the maximum expiratory flow ranged from 0.16 to 0.34 *f*_TLC_ s^−1^.

### Lung compliance

Figure 1B shows selected end-expiratory and end-inspiratory points of zero flow during a spontaneous breath (a and b), which were used to determine the dynamic compliance (C_L_, Figure 1B). A subset (*n* = 393 breaths) of all breaths had reliable trans-pulmonary data, and were used to calculate C_L_. The average (±*SD*) C_L_ was 0.31 ± 0.12 L cmH_2_O^−1^ (*n* = 4 animals, Table 1). As C_L_ varies with lung size (Stahl, 1967), the specific lung compliance (sC_L_, cmH_2_O^−1^) was computed by dividing C_L_ by the minimum air volume (MAV), which was estimated to be 7% of TLC_est_ based on previous experiments with excised lungs (Fahlman et al., 2011). In species where the chest compliance does not limit lung compression, the MAV can be used as an estimate of the residual volume (RV), and also of functional residual capacity (FRC, Fahlman et al., 2014a). Pinnipeds have been show to have very high chest wall compliance (i.e., a chest wall that does not resist compression, Leith, 1976; Fahlman et al., 2014a). Thus, we assumed that the elastic recoil of the chest does not limit emptying of the lung in the sea lions in this study. The estimated MAV ranged from 0.7 to 1.7 L and the average sC_*L*_ was 0.41 ± 0.11 cmH_2_O^−1^.

### Respiratory gas composition and metabolic rate

We measured both end-expiratory O_2_ and CO_2_ only in May. In November the CO2 analyzer was not working properly, so we only report O_2_ and CO_2_ results from 4 sea lions measured in May.

The average (±*SD, n* = 4, Table 1) end-expiratory CO_2_, $\dot{V}$*CO*_2_, and RQ were 7.4 ± 0.4%, 0.78 ± 0.32 L CO_2_ min^−1^, and 0.78 ± 0.05, respectively. The average (±*SD, n* = 4) end-expired O_2_, $\dot{V}$*O*_2_, and s$\dot{V}$*O*_2_ were 13.1 ± 1.0 %, 1.02 ± 0.33 L O_2_ min^−1^, 530 mL O_2_ min^−1^ kg^−1^, respectively. The average $\dot{V}$*O*_2_ was significantly higher in the male sea lion (1.35 ± 0.23 L O_2_ min^−1^) as compared with females (0.80 ± 0.10 L O_2_ min^−1^, LRT = 32.6, *P* < 0.001, Table 1), but for s$\dot{V}$*O*_2_ there were no differences between gender (Welch *t*-test, *t*-value = 0.71, *P* > 0.5, 53.0 ± 15.2 mL O_2_ min^−1^ kg^−1^). While there were no differences between gender, additional data are required to confirm this hypothesis. The Kleiber ratio was estimated by dividing the observed metabolic rate (Table 1) by the predicted basal metabolic rate (BMR, L O_2_ min^−1^) using Kleiber's equation (BMR = 0.00993 $M_{\text{b}}^{0.75}$; Kleiber, 1961). The Kleiber ration ranged from 1.71 to 3.67, and the overall average was 3.1 for all data.

**Figure 5:**
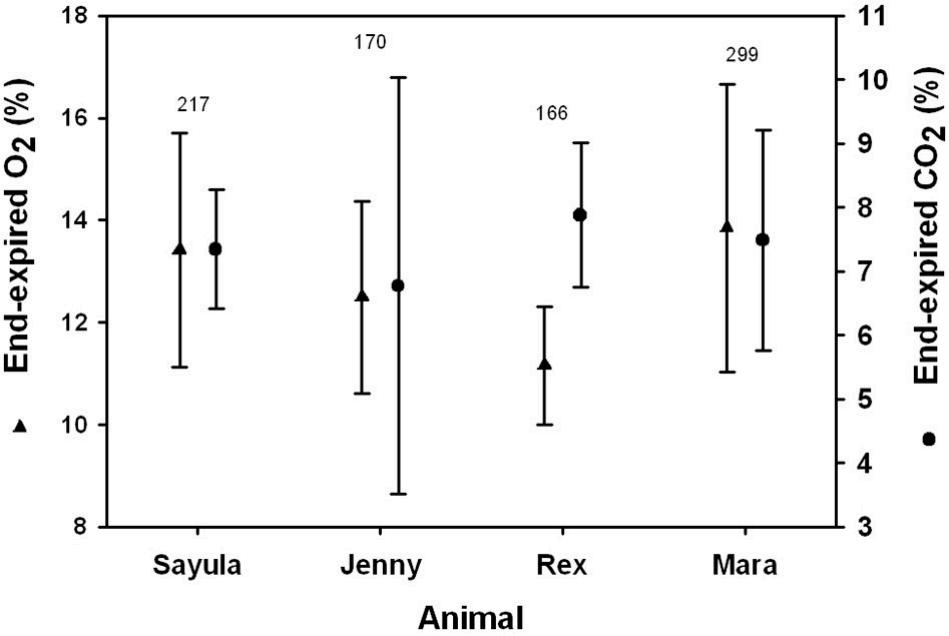
**Average (±*SD*) inspiratory and expiratory tidal volumes for four Patagonia sea lions**.

The average end-expiratory O_2_ was 12.6 ± 1.1 % and did not differ between genders (*t*-value = 2.9, Wald's test, *P* > 0.05, Figure 5). The end-expiratory O_2_ decreased with increasing tidal volume (*t*-value = 13.5, Wald's test, *P* < 0.01). The end-expiratory O_2_ ranged from a minimum of 7.1 % for an inspiratory tidal volume of 5.4 L (*M*_b_ = 91 kg) to 19.6 % for a tidal volume of 1.7 L (*M*_b_ = 97.5 kg).

## Discussion

This is the first study having measured respiratory physiology and metabolic rate in Patagonia sea lions during voluntary breathing. Physiological studies on trained animals that participate voluntarily are useful as they minimize the stress involved as compared with studies using forced restraint. The results suggest that the sC_L_ is similar to those measured in anesthetized sea lions, and significantly higher as compared with humans (Fahlman et al., 2014a). In addition, the measured tidal volumes during voluntary breaths are higher than those predicted in terrestrial mammals, and the respiratory flow similar to those reported in California sea lions (Kerem et al., 1975; Matthews, 1977). The estimated resting metabolic rates were similar to those measured in Steller sea lions and California sea lions in water (Hurley and Costa, 2001; Fahlman et al., 2008, 2013) and Steller sea lions in air (Rosen and Trites, 2000). Although the mass specific metabolic rate appeared similar between females and the single male sea lion, data from additional males are required to address this question meaningfully. Thus, the results presented here are likely to represent baseline metabolic and lung function values for healthy Patagonia sea lions.

Previous studies have measured respiratory physiology in a number of marine mammals, ranging from work on restrained, semi-restrained, and trained animals participating voluntarily (Irving et al., 1941; Scholander and Irving, 1941; Olsen et al., 1969; Kooyman et al., 1971; Kerem et al., 1975; Matthews, 1977; Gallivan and Best, 1980; Gallivan, 1981; Kooyman and Cornell, 1981; Reed et al., 1994, 2000; Fahlman et al., 2015). An important aspect for studies on trained animals is that the individual subject participates voluntarily. This helps minimize the potential stress associated with restraint, and exposure to novel environments, or situations. However, for studies on trained animals the influence of the trainer cannot be discarded. For this reason, it is important that the study subjects undergo desensitization to assure that the experiment minimally affect the physiological variables being measured. In the current study, the respiratory frequency during the research trials was compared with focal observations of each animal while they were undisturbed. These data confirm that the experimental procedure did not alter the breathing frequency of the animals. Another important aspect to consider is that the measurements were made in sternal recumbancy. There may be differences in respiratory function and mechanics in this position as compared to animals in water, due to the compression on the chest. Thus, this is an important aspect to investigate in future studies.

The measured tidal volumes increased with size (Figure 3), and following correction the mass-specific tidal volume ranged from 47 to 73% of TLC_est_. This mass-specific tidal volume is significantly higher as compared with the estimated value of 14% in terrestrial mammals (Stahl, 1967). Mortola and Sequin (2009) assembled published data on VT for marine mammals and the equation predicting tidal volume was VT = 22 $M_{\text{b}}^{1.01}$, where VT is tidal volume in mL, and *M*_b_ the body mass in kg. The results in the current study agree well with the tidal volume estimates from this equation, and are consistent with the general observation by others that marine mammal take deeper breaths (Olsen et al., 1969; Kooyman, 1973; Matthews, 1977; Kooyman and Cornell, 1981; Fahlman et al., 2015) and have a reduced breathing frequency (Mortola and Limoges, 2006) as compared with terrestrial mammals. The marine mammal breathing strategy seems to be conserved evolutionary, expressed at birth, and maintained in both fully aquatic (cetaceans) and semi-aquatic (pinnipeds) marine mammals (Mortola and Limoges, 2006). It has been suggested that increased buoyancy at the water surface may be one possible explanation for this breathing strategy (Mortola and Limoges, 2006). There is human clinical interest in this breathing pattern, called airway pressure release ventilation (APRV), as it may be useful to improve gas exchange during anesthesia (Daoud et al., 2012). Thus, this breathing pattern may help prevent a substantial increase in alveolar CO_2_ (P_A_CO_2_) (Mortola and Sequin, 2009) during intermittent/apneustic breathing. Breath-by-breath respirometry provides a useful tool to study the possible evolutionary advantages of the aquatic breathing strategy, with an extended inspiratory pause, as compared with terrestrial breathing pattern, and may improve knowledge how marine mammals manage gases during diving.

Breath-by-breath respirometry provides additional advantages from conventional flow-through respirometry, but is also logistically challenging. For example, the O_2_ uptake and CO_2_ produced can be estimated for each breath, making it possible to determine how blood gases change following exercise (Fahlman et al., 2016), or diving (Kooyman et al., 1973; Reed et al., 1994, 2000). To estimate the O_2_ taken up on a breath-by-breath basis requires matching of respiratory flow and expired gas content. This is challenging if the respiratory flow is fast and the breath duration short. In a previous study, we managed to reliably estimate O_2_ uptake and CO_2_ production per breath in bottlenose dolphins (Fahlman et al., 2015). As the breath duration is 2–4 times as long (Figure 2), and the respiratory flow an order of magnitude slower (Figure 4) in the sea lions as compared with the dolphins, the O_2_ uptake should be accurately estimated. In addition, the estimated mass-specific resting metabolic rates (sRMR: 4.1–10.5 mL O_2_ min^−1^ kg^−1^) were similar to those measured in Steller sea lions and California sea lions in water (Steller sea lions: 7.4–9.2 mL O_2_ min^−1^ kg^−1^; California sea lions: 5.7–10.4 mL O_2_ min^−1^ kg^−1^, Hurley and Costa, 2001; Fahlman et al., 2008, 2013) and Steller sea lions in air (3.0–9.5 mL O_2_ min^−1^ kg^−1^, Rosen and Trites, 2000). As both the measured tidal volumes and metabolic rates are within the range of those measured in other pinnipeds of similar size, the reported data likely represent valid estimates for this species. Thus, using this system of gas analyzer and pneumotachometer we can accurately estimate breath-by-breath O_2_ uptake. In addition, the fast response gas analyzer allows us to estimate end-expiratory gas content, or end-tidal gas composition. This may provide for interesting insights into respiratory health, and be a useful non-invasive method to assess lung disease in marine mammals (Van Elk et al., 2001). Despite its challenges, breath-by-breath respirometry provides data that have wide applicability for many research fields, and has previously been used to answer interesting questions in marine mammal eco-physiology (Reed et al., 1994, 2000). Improved understanding of the physiological limitations of marine species is vital to make accurate predictions how they may respond to changes in the environment. This will further enhance conservation and management efforts to reduce the impact on marine species.

During diving marine vertebrates incur an O_2_ debt that is paid back at the end of the dive (Scholander, 1940). When marine mammals dive repeatedly with only a short surface interval (a diving bout), data indicate that the O_2_ debt is not repaid between dives, but that they dive again before they completely replenish the O_2_ stores (Fahlman et al., 2008). The rate of gas exchange is governed by the partial pressure gradient, and this is largest when the animal returns to the surface to breathe. In addition, as the O_2_ dissociation curve is sigmoidal the most efficient uptake is on the steep portion of the curve. As the partial pressure gradient decreases with each breath, the amount of O_2_ taken up therefore decreases with time. Thus, by not completely restoring the O_2_ the animals minimize time spent at the surface and maximize time at depth (Kramer, 1988; Thompson and Fedak, 2001). However, this diving strategy is likely to cause gradual accumulation of CO_2_ and/or N_2_ with each subsequent dive, eventually forcing a prolonged surface interval or a period of very short and shallow dives (Scholander, 1940; Fahlman et al., 2009; Hooker et al., 2009). As a result, the management of O_2_ may govern the duration of each dive and surface interval, while accumulation of CO_2_ and/or N_2_ may determine the duration of a bout. Changes in dive behavior, as may occur with changes in prey availability and/or environmental change may alter the accumulation of CO_2_ and N_2_, and thereby the foraging efficiency. Breath-by-breath analysis of gas exchange is a powerful tool to further understand how marine mammals manage gases during diving and has provided interesting results that provide information about what physiological variables that may limit diving (Reed et al., 1994, 2000; Boutilier et al., 2001). Studies using such methods would be useful to assess how changes in dive behavior and physiology may cause animals to exceed physiological limits that may cause gas bubble disease (Bostrom et al., 2008; Fahlman et al., 2009, 2014b; Fitz-Clarke, 2009; Hooker et al., 2009).

Scholander suggested that high chest and alveolar compliance, and low compliance of the conducting airways should help to minimize gas exchange during diving and thereby the risk of decompression sickness (Scholander, 1940). Denison et al. (1971) showed that the cartilaginous reinforcement of the conducting airways helped the alveoli to empty more completely in California sea lions as compared with the dog. This higher sCL as compared with terrestrial mammals have also been reported in a range of other marine mammals (Olsen et al., 1969; Leith, 1976; Bergey and Baier, 1987; Fahlman et al., 2014a, 2015) and the data in the Patagonia sea lion agree. The increased understanding of the mechanical properties of the respiratory system may help us better understand how marine mammals manage gases during diving, avoid pressure related problems, and provide clues how they perform extended dives to great depths. Modeling efforts may help increase the understanding how marine mammals respond to environmental change, but model results are affected by the quality of the data and the accuracy of the model assumptions. For example, a number of studies have attempted to model how the interaction between behavior, physiology, and environmental pressure affect the risk of decompression sickness during breath-hold diving (Houser et al., 2001; Zimmer and Tyack, 2007; Fahlman et al., 2009, 2014b; Fitz-Clarke, 2009; Hooker et al., 2009). The theoretical work performed on marine mammals used parameter estimates from terrestrial species or from marine mammals species that may be distantly related, i.e., Weddell seal vs. beaked whale. In a recent study it was shown that when modeling gas dynamics in California sea lions using species-specific parameters, the model output was significantly different as to when using parameters from terrestrial species (Hodanbosi et al., 2016). This highlights the importance of studies to better understand physiological limitations that may impact how environmental change limit survival of marine mammals.

The esophageal balloon catheter provides information of the mechanical properties of respiration (Fahlman et al., 2015). For example, in Figure 1B there was little or no change in the transpulmonary pressure during exhalation, suggesting that this phase is passive and mostly governed by the elastic recoil of the lung and chest down to FRC. At the beginning of the inhalation the transpulmonary pressure decreases which indicates that the respiratory muscles help with the inspiratory phase. Similar results were seen during voluntary breaths in bottlenose dolphins (Fahlman et al., 2015). However, in the bottlenose dolphin both the expiratory and inspiratory phases were active during maximal efforts. We currently do not have any result from sea lions performing maximal respiratory efforts but it is likely that in sea lions the respiratory muscles may help during exhalation in this species too.

### Summary

We collected respiratory gas composition and flow from five Patagonia sea lions to assess respiratory physiology and metabolism. Our results suggest that the mass-specific tidal volume and sC_*L*_ were significantly higher as compared to terrestrial mammals, but similar to other marine mammals. The respiratory flow and breath durations were significantly lower and longer, respectively, as compared with bottlenose dolphins. The mass-corrected metabolic rate was between 1.71 and 3.67 higher than the basal metabolic rate estimated by Kleiber's allometric equation for basal metabolic rate (Kleiber, 1961). Consequently, the resting metabolic rate in Patagonia sea lions is substantially higher than the predicted basal metabolic rate, but this range is similar to other pinnipeds species. We conclude that the respiratory physiology and energy requirements for Patagonia sea lions are similar to those of other Pinnipeds.

## Author contributions

AF conceived of the study, designed the experiments, collected and analyzed the data, carried out the statistical analysis, and drafted the paper; JM participated in the design of the study and was in charge of animal training.

## Funding

Funding for this project was provided by the Office of Naval Research (ONR YIP Award # N-000141410563) and by Dolphin Adventure and Oceanografic Foundation.

### Conflict of interest statement

The authors declare that the research was conducted in the absence of any commercial or financial relationships that could be construed as a potential conflict of interest.
