# Corrigendum: Respiratory Function in Voluntary Participating Patagonia Sea Lions (*Otaria flavescens*) in Sternal Recumbency

**DOI:** 10.3389/fphys.2016.00670

**Published:** 2017-01-05

**Authors:** Andreas Fahlman, Johnny Madigan

**Affiliations:** ^1^Fundación Oceanografic de la Comunidad ValencianaValencia, Spain; ^2^Department of Life Sciences, Texas A&M University-Corpus ChristiCorpus Christi, TX, USA; ^3^Dolphin AdventureNuevo Vallarta, Mexico

**Keywords:** tidal volume, breath duration, diving physiology, respiratory flow rate, lung compliance

1. A reference to the metabolic cost in Patagonia sea lions is missing in the first paragraph of the discussion. The following sentence should be corrected: The estimated resting metabolic rates were similar to those measured in Steller sea lions and California sea lions in water (Hurley and Costa, 2001; Fahlman et al., 2008, 2013) and Steller sea lions in air (Rosen and Trites, 2000).

The correct sentence should be: The estimated resting metabolic rates were similar to those measured in Patagonia sea lions, Steller sea lions, and California sea lions in water (Hurley and Costa, 2001; Fahlman et al., 2008, 2013; Dassis et al., 2012) and Patagonia and Steller sea lions in air (Rosen and Trites, 2000; Dassis et al., 2012).

2. The following sentence in the discussion should include the previous study on Patagonia sea lions: In addition, the estimated mass-specific resting metabolic rates (sRMR: 4.1–10.5 mL O_2_ min^−1^ kg^−1^) were similar to those measured in Steller sea lions and California sea lions in water (Steller sea lions: 7.4–9.2 mL O_2_ min^−1^ kg^−1^; California sea lions: 5.7–10.4 mL O_2_ min^−1^ kg^−1^, Hurley and Costa, 2001; Fahlman et al., 2008, 2013) and Steller sea lions in air (3.0–9.5 mL O_2_ min^−1^ kg^−1^, Rosen and Trites, 2000).

The sentence including previous study should be:

In addition, the estimated mass-specific resting metabolic rates (sRMR: 4.1–10.5 mL O_2_ min^−1^ kg^−1^) were similar to those measured in Patagonia sea lions, Steller sea lions and California sea lions in water (Patagonia sea lions: 9.0 mL O_2_ min^−1^ kg^−1^; Steller sea lions: 7.4–9.2 mL O_2_ min^−1^ kg^−1^; California sea lions: 5.7–10.4 mL O_2_ min^−1^ kg^−1^; Hurley and Costa, 2001; Fahlman et al., 2008; Dassis et al., 2012; Fahlman et al., 2013) and Patagonia sea lions and Steller sea lions in air (Patagonia sea lions: 4.3–9.1; Steller sea lions: 3.0–9.5 mL O_2_ min^−1^ kg^−1^, Rosen and Trites, 2000; Dassis et al., 2012).

3. The species studied is also commonly called South American sea lion or Southern sea lion.

4. A typo was detected in the reported mass-specific metabolic rate (s $\overset{\cdot }{V}$O_2_) and the reported value for s $\overset{\cdot }{V}$O_2_ should be 53 mL O_2_ • min^−1^ • kg^−0.6^ or 8.6 ± 1.9 mL O_2_ • min^−1^ • kg^−1^.

## Conflict of interest statement

The authors declare that the research was conducted in the absence of any commercial or financial relationships that could be construed as a potential conflict of interest.
